# Non-vitamin K antagonist oral anticoagulants in venous thromboembolism patients: a meta-analysis of real-world studies

**DOI:** 10.1186/s12872-022-02550-8

**Published:** 2022-03-14

**Authors:** Zhi-Yan Liu, Han-Xu Zhang, Ling-Yue Ma, Guang-Yan Mu, Qiu-Fen Xie, Shuang Zhou, Zi-Ning Wang, Zhe Wang, Kun Hu, Qian Xiang, Yi-Min Cui

**Affiliations:** 1 Department of Pharmacy, Peking University First Hospital, No. 6, Dahongluochang Street, Xicheng District, Beijing, 100034 China; 2 School of Pharmaceutical Sciences, Peking University Health Science Center, Beijing, China

**Keywords:** NOACs, VTE, Clinical outcomes, Direct comparison, Meta-analysis

## Abstract

**Background:**

The real-world studies on recurrent venous thromboembolism (VTE) and bleeding events of non-vitamin K antagonist oral anticoagulants (NOACs) in VTE patients have reported conflicting findings. Our study aimed to provide the direct comparison evidence of different NOACs for VTE patients in clinical practice settings.

**Methods:**

Search of the medical literature was conducted using PubMed, Web of Science, EMBASE, Clinical Trials.gov, and the Cochrane Library from inception to March 22, 2021. Among the 19,996 citations retrieved, a total of 63,144 patients from 6 studies were analyzed. Clinical outcomes included recurrent VTE, death, and different bleeding events.

**Results:**

Adjusted hazard ratio (HR) analysis suggested that apixaban had significant lower bleeding riskthan rivaroxaban (major, minor and any bleeding: HR = 0.61, 0.56, 0.70; *p* = 0.008, < 0.0001, 0.006, respectively), but no statistics difference found in recurrent VTE events (HR = 1.02, 95% confidence interval (CI) 0.71–1.47, *p* = 0.93). There was no significant difference of major bleeding between dabigatran and rivaroxaban (odds ratios (OR) = 0.41, 95% CI 0.09–1.90, *p* = 0.25), apixaban and dabigatran (OR 0.64, 95% CI 0.15–2.72, *p* = 0.83). No significant difference was found in the comparison of edoxaban and other NOACs in VTE recurrence, major bleeding and composite outcome.

**Conclusions:**

In the prevention of bleeding events, apixaban was associated with a lower risk than rivaroxaban, but equivalent efficacy for different NOACs in prevention of recurrent VTE. Evidence generated from the meta-analysis based on real-world data can help to guide selection between apixaban and rivaroxaban in routine clinical practice.

*Trial registration*: This systematic review and meta-analysis were conducted and reported according to the Preferred Reporting Items for Systematic Reviews and Meta-analysis and Meta-analysis of Observational Studies in Epidemiology statements and was registered with PROSPERO (CRD42019140553).

**Supplementary Information:**

The online version contains supplementary material available at 10.1186/s12872-022-02550-8.

## Background

Venous thromboembolism (VTE) comprises both deep vein thrombosis (DVT) and pulmonary embolism (PE) [1]. VTE is often overlooked, and results in long-term complications including post thrombotic syndrome (PTS) for DVT, post pulmonary embolism syndrome and chronic thromboembolic pulmonary [2]. 30% of VTE individuals will develop a recurrence within 10 years of their initial event [3].

The first 3–6 months of anticoagulant treatment for VTE is generally viewed as active treatment of the initial thrombosis. Continuing anticoagulation treatment can reduce the risk of recurrent VTE but might increase bleeding risk [4]. Heparin, the low-molecular-weight heparin (LMWHs), fondaparinux and the non-vitamin K antagonist oral anticoagulants (NOACs) are the only agents approved by the US Food and Drug Administration (FDA) recommended for the acute treatment phase. NOACs and warfarin are anticoagulation options for the long-term and extended treatment phases. The NOACs, which include the factor Xa inhibitors rivaroxaban, apixaban, and edoxaban and the direct thrombin inhibitor dabigatran, are equally efficacious for the initial treatment of VTE as compared with vitamin K antagonists and superior to placebo or aspirin for secondary VTE prevention [5–7]. An analysis of data from Danish nationwide registries in 2016 reported that 70% of patients with VTE initiated rivaroxaban, 16% initiated apixaban, 2% initiated vitamin K antagonists (VKAs), and 2% initiated dabigatran [7].

With the licensing and availability of NOACs, data are needed on their comparative safety and effective profile in many countries to guide their decision-making for patients and clinicians. The clinical trial of EINSTEIN (Oral rivaroxaban for symptomatic venous thromboembolism and oral rivaroxaban for symptomatic pulmonary embolism) and AMPLIFY (Oral apixaban for the treatment of acute venous thromboembolism) that established the safety and efficacy of rivaroxaban and apixaban were heterogeneous [8–10]. The absolute rate of recurrent VTE and bleeding were 2.1%, 1.0–1.7% in the EINSTEIN studies and 2.3%, 0.6% in the AMPLIFY study [8–10]. In contrast, Cohen et al. found that apixaban was associated with a significantly lower risk of clinically relevant non-major bleeding or major bleeding (hazard ratio (HR) 0.47, 95% CI (confidence interval) 0.36–0.61) in the network meta-analysis [11]. Head-to-head comparisons of clinical trials between different NOACs have not been done in patients with VTE [12]. Other evidence that bleeding risk may be greater with rivaroxaban than apixaban comes from network meta-analysis of phase III RCTs, which provided indirect evidence of higher major bleeding risk for rivaroxaban versus apixaban, though precision was poor [13, 14]. At present, one meta-analysis that directly compares rivaroxaban with apixaban indicated that apixaban showed equivalent efficacy in prevention of recurrent VTE but decreased risk of major and minor bleeding events compared with rivaroxaban [15].

Observational studies that found an increased menstrual bleeding with rivaroxaban versus apixaban [16, 17], while other observational study with 8187 VTE patients suggested no significant differences between rivaroxaban or apixaban in the risk of all-cause mortality, recurrent VTE, or hospitalized bleeding [18]. Large observational data sets may provide an opportunity to study comparative different NOACs to inform the choice among NOACs. Inconsistency based on observational research results, thus, the aim of our systematic review and meta-analysis including: (1) compare clinical outcomes between dabigatran, rivaroxaban, apixaban, and edoxaban directly in patients with VTE; (2) provide direct evidence for patients and doctors in choosing different NOACs using observational data sets.

## Methods

### Search strategy and study selection

Search of the medical literature was conducted using PubMed, Web of Science, EMBASE, Clinical Trials.gov, and the Cochrane Library from inception to March 22, 2021. A study was included for analysis if it met the following pre-specified criteria: (1) patients had a diagnosis of VTE, treating with NOACs (including apixaban or dabigatran or rivaroxaban or edoxaban); (2) clinical outcomes [systemic embolism (SE), VTE, bleeding or death events] were directly compared between different NOACs; (3) Clinical trials, cohort studies and case–control studies were included in the analysis; (4) Only English language and full-text articles were considered. The exclusion criteria were: (1) clinical outcomes without needed direct comparison data; (2) duplicate reports and case reports were excluded.

Citations recalled were initially screened with title and abstract (Zhiyan Liu and Hanxu Zhang), and then two investigators retrieved and assessed the full texts of potentially relevant studies for their eligibility. Any disagreement between investigators was resolved by consensus.

### Outcomes assessment

Clinical outcomes included recurrent VTE, death, and different bleeding events. Different bleeding events included major bleeding, intracranial bleeding, gastrointestinal (GI) bleeding, hospitalized bleeding and minor bleeding. Definition of clinical outcomes in included studies is reported in Additional file 2: Table S1.

### Data extraction and quality assessment

Details were extracted with pre-specified table on the study details, patient characteristics, drug regimes, clinical outcomes, and follow-up time, among others. The Newcastle–Ottawa Scale (NOS) was used to assess the methodological quality of included studies [19, 20]. Natural logarithms of reported hazard ratios and corresponding standard errors were extracted from studies where available. Each study with NOS scores ≥ 7 was considered as a high-quality study, whereas studies with NOS scores < 7 were considered as low-quality studies. Assessment was performed independently by two investigators, with disagreements resolved by discussion.

### Data synthesis and statistical analyses

Data were pooled using a random or fixed effects model to obtain a more conservative estimate of clinical outcomes of different NOACs. Measures of association in the form of odds ratios (OR) or adjusted hazard ratio (HR) were pooled, and 95% confidence interval (CI) were selected as the summary statistic. I^2^ statistic and p-value for Q statistics were used to estimate the percentage of variability across studies that was attributable to heterogeneity. If I^2^ > 50%, it indicates that there is high heterogeneity, and the causes of heterogeneity need to be analyzed. In case of clinical heterogeneity, subgroup analysis was performed to eliminate heterogeneity or descriptive analysis was performed; If there was no clinical heterogeneity but only statistical heterogeneity, the random effect model was used for analysis. If I^2^ ≤ 50%, the heterogeneity is acceptable, and the fixed effect model is used for analysis.

Studies reported adjusted hazard ratio (HR) results were adjusted by multiple factors in patient characteristics (including age, gender, region, duration of follow-up, baseline bleeding status, baseline stroke status, baseline comorbidities, and baseline co-medication usage status). The p-value for statistical significance was 0.05 in all cases, except the test for heterogeneity, in which the level was set at 0.10.

Review Manager Version 5.3 (Rev Man for Windows, the Nordic Cochrane Centre, Copenhagen, Denmark) was used to generate forest plots of pooled ORs for primary outcomes with 95% CI. Both pooled HR analysis, and publication bias assessment were performed using the STATA software (version 15.3; Stata Corporation, College Station, TX, the USA).

This systematic review and meta-analysis were conducted and reported according to the Preferred Reporting Items for Systematic Reviews and Meta-analysis and Meta-analysis of Observational Studies in Epidemiology statements and was registered with PROSPERO (CRD42019140553, the Date of registration in PROSPERO was 19 September 2019, the article is a part of the study; First search time for this study was March, 2020, we conducted a supplementary search in 2021).

## Results

**Fig. 1:**
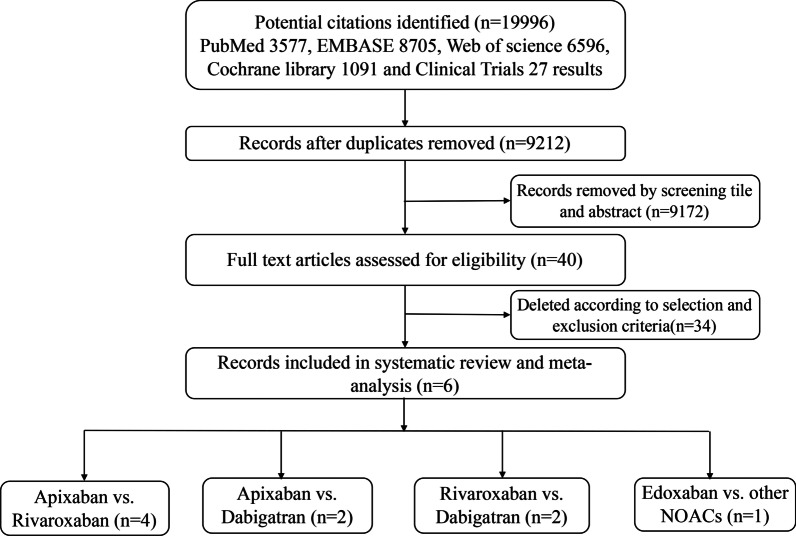
A flow diagram of study identification and selection

40 articles were thought to be potentially eligible for inclusion after reviewing titles and abstracts among identified 19,996 citations. Finally, 63,144 VTE patients from 6 observational studies [18, 21–25] reporting the effectiveness and safety outcomes were included in the meta-analysis. All patients were Caucasian. The follow-up time for clinical outcomes ranged from 3 to 6 months. Two registered random controlled studies were found: the COBRA (NCT03266783) and CANVAS (NCT02744092) trials, which were still recruiting participants. A flow diagram of study identification and selection is shown in Fig. 1. For different research reports from the same database population, we choose the research with large time span and large population. Characteristics of the included studies are summarized in Table 1a, b. On quality assessment, 2 studies had a score of nine or eight, 2 studies had a score of seven, and 2 studies had a score of six.

**Table 1:** (a) Characteristics of studies included in the meta-analysis, (b) drug regimens in each study included

Study	Study design	Data sources	Numbers	Patients	Therapy regimen	Follow-up times	Outcomes	NOS scales
*(a)*								
Dawwas GK, 2018 [19]	Retrospective	The Truven Health Market Scan commercial and Medicare Supplement claims databases in the USA between Jan 1, 2014, and Dec 31, 2016	15,254	VTE	Rivaroxaban, apixaban	3 months	Recurrent VTE, MB	8
Bott-Kitslaar DM, 2019 [20]	Prospective	The Mayo Thrombophilia Clinic Registry between Mar 1, 2013, and Jan 30, 2018	600	VTE	Rivaroxaban, apixaban	3 months	Recurrent VTE, MB, CRNMB	9
Davis DO, 2017 [21]	Retrospective	Ochsner Medical Center from January 1, 2013, through December 31, 2015	37	VTE	Dabigatran, rivaroxaban, apixaban	6 months	Recurrent VTE, bleeding	6
López-Núñez JJ, 2019 [22]	Retrospective	Data in the RIETE registry Jan 2013 to Apr 2018	1298	VTE	Dabigatran, rivaroxaban, apixaban, edoxaban	3 months	Recurrent VTE, MB	6
Lutseya PL, 2019 [23]	Retrospective	Market Scan data warehouse (Truven Health Analytics) for the time-period from Jan 1, 2011 to Dec 31, 2016	37,768	VTE	Rivaroxaban, apixaban	6 months	Hospitalized bleeding	7
Sindet-Pedersen C, 2018 [18]	Retrospective	Danish nationwide registries from Jan 1, 2015 to Jun 30, 2017	8187	VTE	Rivaroxaban, apixaban	6 months	All-cause mortality, recurrent VTE, hospitalized bleeding	7

Study	Race	Apixaban					Rivaroxaban				
		No	Age	Gender (M, %)	Renal disease (%)	Antiplatelets (%)	No	Age	Gender (M, %)	Renal disease (%)	Antiplatelets (%)
(b)											
Dawwas GK, 2018 [19]	Caucasian	3091	61.6 ± 16.4	49.4	17.1	7.20	12,163	59.9 ± 16.2	49.6	15.3	6.50
Bott-Kitslaar DM, 2019 [20]	Caucasian	302	62.4 ± 14.0	62.3	9.00	22.5	298	58.5 ± 14.2	52.0	4.00	20.5
Davis DO, 2017 [21]	Caucasian	Total age: 69 ± 4.75, Gender: 45.9% male, antiplatelet 27.0%									
López-Núñez JJ, 2019 [22]	Caucasian	Total age: 79 ± 9.9, Gender: 37.0% male, renal disease 41%, antiplatelet 2.0%									
Lutsey PL, 2019 [23]	Caucasian	6786	60.4 ± 16.2	49.6	13.2	7.00	30,982	56.4 ± 15.4	51.8	7.10	5.10
Sindet-Pedersen C, 2018 [18]	Caucasian	1504	70.0 ± 17.8	49.2	3.90	15.2	6683	67.0 ± 17.1	54.7	2.70	12.9

Wysokinski WE, et al. had the same population from the same database with Bott-Kitslaar DM, 2019 [20], thus this study was not included in the mate-analysis. (Wysokinski WE, Houghton DE, Casanegra AI, et al. Comparison of apixaban to rivaroxaban and enoxaparin in acute cancer-associated venous thromboembolism. Am J Hematol. 2019;94(11):1185‐1192. https://doi.org/10.1002/ajh.25604)

*VTE* venous thromboembolism, *SE* systemic embolism, *MB* major bleeding, *GI* gastrointestinal, *CRNMB* clinically relevant nonmajor bleeding, *NOS* Newcastle–Ottawa Scale

### Apixaban versus rivaroxaban

Comparison of rivaroxaban and apixaban was conducted in all included studies [18, 21–25]. Baseline comparison between different two groups is shown in Additional file 3: Table S2. No significant difference of baseline characters was found in comparison (*p* > 0.05). All patients were Caucasian. The total average age was 62.0 ± 4.47 (mean ± standard deviation (SD)) years old, with a 52.3 ± 4.45 percent of males. The mean (± SD) follow-up time for clinical outcomes was 4.50 ± 1.73 months in the comparison.

**Fig. 2:**
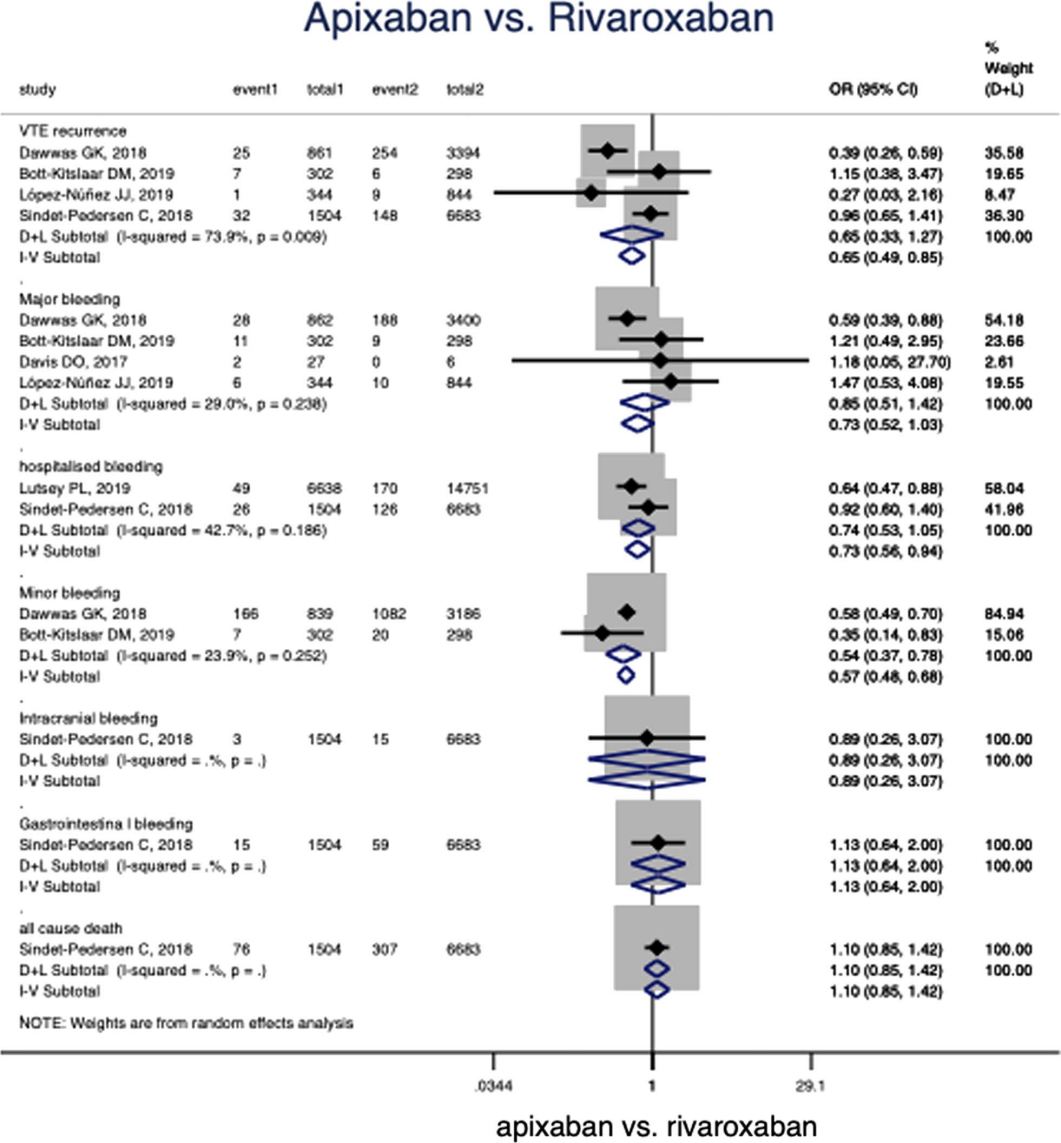
Comparison of apixaban and rivaroxaban in different clinical outcomes

For the recurrent VTE events, no significant difference was found between apixaban (n = 3011) and rivaroxaban (n = 11,219) (OR 0.65, 95% CI 0.33–1.27, *p* = 0.22, Fig. 2). For major, hospitalized, intracrarial, GI bleeding and death events, no difference was found among the two drugs (*p* > 0.05, Fig. 2). However, apixaban showed a 46% lower risk in minor bleeding (OR 0.54 95%CI 0.37–0.78, *p* < 0.001, Fig. 2) than that of rivaroxaban. Adjusted HR was also reported and pooled in the studies. Apixaban had significant lower bleeding (major, minor and any bleeding) risk than rivaroxaban (HR = 0.61, 0.56, 0.70; *p* = 0.008, < 0.0001, 0.006, respectively, Additional file 1: Figure S1). There was no statistics difference between the two groups in recurrent VTE events (HR = 1.02, 95% CI 0.71–1.47, *p* = 0.93, Additional file 1: Figure S1). One study [21] reported death result, showed that no different death risk existed between apixaban and rivaroxaban (HR = 1.11, 95% CI 0.87–1.41, *p* = 0.40, Additional file 1: Figure S1).

### Rivaroxaban versus dabigatran

**Fig. 3:**
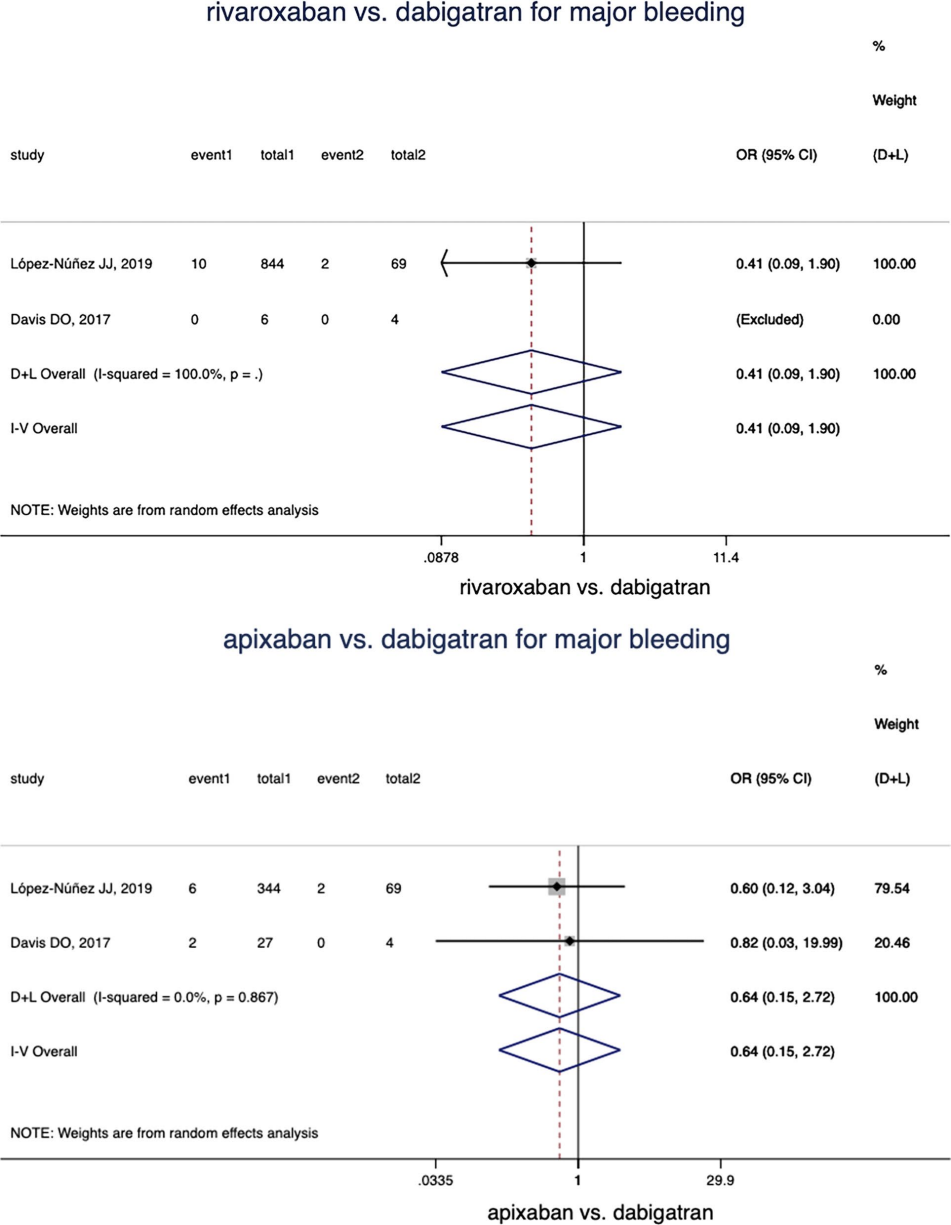
Comparison of dabigatran and rivaroxaban in different clinical outcomes

2 studies involved 923 patients reported the incidence rates of major bleeding were analyzed in comparison of rivaroxaban and dabigatran, without adjusted HR data available. There was no significant difference of major bleeding between dabigatran and rivaroxaban (OR = 0.41, 95% CI 0.09–1.90, *p* = 0.25, Fig. 3). Only one study [23] reported the recurrent VTE outcomes in the direct comparison of dabigatran and rivaroxaban. There are 9 and 0 patients had recurrent VTE among 844 rivaroxaban and 69 dabigatran patients.

### Apixaban versus dabigatran

2 studies involved 444 patients reported the incidence rates of major bleeding were analyzed, without adjusted HR data available. There was no statistically difference between apixaban and dabigatran in major bleeding risk (OR = 0.64, 95% CI 0.15–2.72, *p* = 0.83, Fig. 3). One study [24] reported the recurrent VTE outcomes in the direct comparison of apixaban and dabigatran. There are 1 and 0 patients had recurrent VTE among 344 apixaban and 69 dabigatran.

### Edoxaban versus other NOACs

**Fig. 4:**
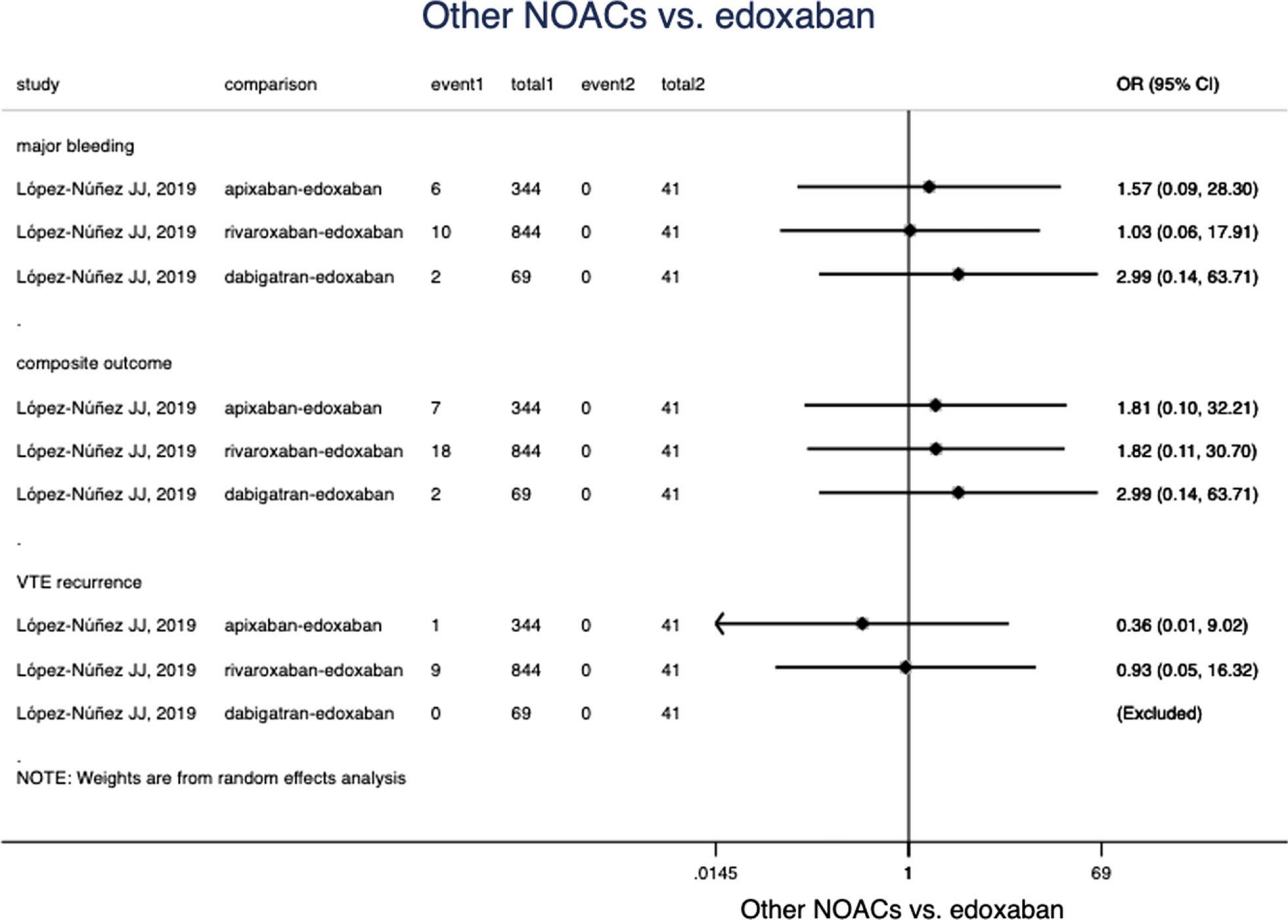
Comparison of edoxaban and other NOACs in different clinical outcomes

Only one study [24] reported comparison of edoxaban with other NOACs. As the limited data available, no significant difference was found in the comparison of edoxaban and other NOACs in VTE recurrence, major bleeding and composite outcome (Fig. 4).

## Sensitivity analyses and publication bias

Formally sensitivity analysis is used when the numbers of included studies are more than 10 eligible publications, thus this study did not conduct the sensitivity analyses. No publication biases were found through the Egger’s test with *p* value < 0.01 in comparison larger than 2 studies. For example, comparison of apixaban and rivaroxaban of recurrent VTE and major bleeding, the *p* value of Egger’s test was 0.394 and 0.385 respectively.

## Discussion

After reviewing titles and abstracts, 63,144 VTE patients from 6 observational studies were included in the meta-analysis. Adjusted HR analysis suggested that apixaban had significant lower bleeding (major, minor and any bleeding) risk than rivaroxaban, but no statistics difference found in recurrent VTE events. There was no significant difference of major bleeding between dabigatran and rivaroxaban, apixaban and dabigatran. No significant difference was found in the comparison of edoxaban and other NOACs in VTE recurrence, major bleeding and composite outcome.

VTE is a common and potentially fatal disease, and the estimated a first acute VTE incidence is 0.7–1.4 per 1000 person-years [26, 27]. In addition, the socioeconomic effect of VTE is significant, with an annual cost ranging from $13.5 billion to $27.2 billion in the United States [28]. Over the past decade, NOACs have been widely used in clinical and were recommended by the 2016 American College of Chest Physicians and 2014 and 2017 European Society of Cardiology guidelines for VTE [12, 29, 30]. NOACs have several advantages over vitamin K antagonists, such as a rapid onset of action and predictable pharmacokinetic profile. This allows for simplified drug administration in a standardized dose and avoids the need for laboratory monitoring and dose adjustments [31]. Without direct comparison of NOACs with one another in clinical trials, the choice for one drug over another are based on different treatment regimens, patient characteristics, and patient preference [31].

Recurrent VTE and bleeding events are the most feared complication in patients receiving anticoagulant therapy for VTE. Therefore, the clinical outcomes of different drugs are also an important factor in drug selection. Cohen et al. [32] reported no difference in the risk of recurrent VTE between apixaban and rivaroxaban by indirectly assessed the efficacy and safety of NOACs (by comparing apixaban and rivaroxaban against standard therapy). Another meta-analysis [13] found no significant difference between apixaban and rivaroxaban (RR = 0.57, 95% CI 0.29–1.15) in the risk of recurrent VTE. These results are in line with our findings in the direct comparison. An observational study directly comparing the efficacy and safety of NOACs used for the treatment of VTE reported that apixaban and rivaroxaban therapy was associated with similar rates of VTE recurrence and major bleeding, with a lower rate of minor with apixaban [22]. However, another observational study [21] found that the use of apixaban compared with rivaroxaban was associated with decreased risk of recurrent VTE (HR = 0.37, 95% CI 0.24–0.55, *p* < 0.0001) and major bleeding events (HR = 0.54, 95% CI 0.37–0.82, *p* = 0.0031) in the multivariable Cox regression models. Results from two randomized controlled trials comparing apixaban and rivaroxaban head to head, the COBRA (NCT03266783) and CANVAS (NCT02744092) trials, which are still recruiting participants, are awaited. Before these results become available, evidence generated from the meta-analysis based on real-world data can help to guide selection between apixaban and rivaroxaban in routine clinical practice.

Compared with the published meta that directly compares rivaroxaban with apixaban [15], this study added comparison of dabigatran and rivaroxaban, apixaban and dabigatran, edoxaban and other NOACs. And this study suggested that no difference found in different NOACs, but lower bleeding (major, minor and any bleeding) risk of apixaban than rivaroxaban.

Our study has several strengths and limitations. First, this is a meta-analysis assessment, based on data from routine clinical practice of clinical outcomes of different NOACs in patients with VTE. Second, in addition to the comparison of the crude data, we also carried out a pooled analysis of the adjusted HR data analysis. Adjusted HR results were adjusted by multiple factors in patient characteristics. Therefore, the results of the analysis are more reliable than those of conventional observational studies, excluding the influence of some confounding factors. Limitations of this study include the limited studies and relatively small sample size of patients involved in the comparison of dabigatran with other NOACs. Furthermore, no specific dose groups were distinguished in drug comparison of included studies, so comparison between specific doses could not be obtained. In addition, we restricted our search to English language, we might miss some studies.

## Conclusion

Our study is unique and distinguished by its ability to directly compare multiple NOACs with 63,144 VTE patients. In the prevention of bleeding events, apixaban was associated with a lower risk than rivaroxaban, but equivalent efficacy for different NOACs in prevention of recurrent VTE. Evidence generated from the meta-analysis based on real-world data can help to guide selection between apixaban and rivaroxaban in routine clinical practice.

## Supplementary Information

**Additional file 1**. **Figure S1.** Comparison of apixaban and rivaroxaban in different clinical outcomes.

**Additional file 2**. Definition of clinical outcomes in the study.

**Additional file 3**. **Table S2.** Baseline comparison between different two groups.

## Data Availability

All data generated or analysed during this study are included in this published article. Zhiyan Liu (email liuzhiyan09@163.com) can be contacted if someone wants to request the data from this study.

## Abbreviations

CI: Confidence interval
DVT: Deep vein thrombosis
GI: Gastrointestinal
HR: Hazard ratio
LMWHs: Low-molecular-weight heparin
NOACs: Non-vitamin K antagonist oral anticoagulants
NOS: Newcastle–Ottawa Scale
VTE: Venous thromboembolism
OR: Odds ratios
PE: Pulmonary embolism
PTS: Post thrombotic syndrome
SE: Systemic embolism
SD: Standard deviation
VKAs: Vitamin K antagonists
