# Supplementary figures and images for: Non-vitamin K antagonist oral anticoagulants in venous thromboembolism patients: a meta-analysis of real-world studies
Source: BMC Cardiovasc Disord. 2022 Mar 14;22:105. doi: 10.1186/s12872-022-02550-8 (PMC8922817; doi:10.1186/s12872-022-02550-8)

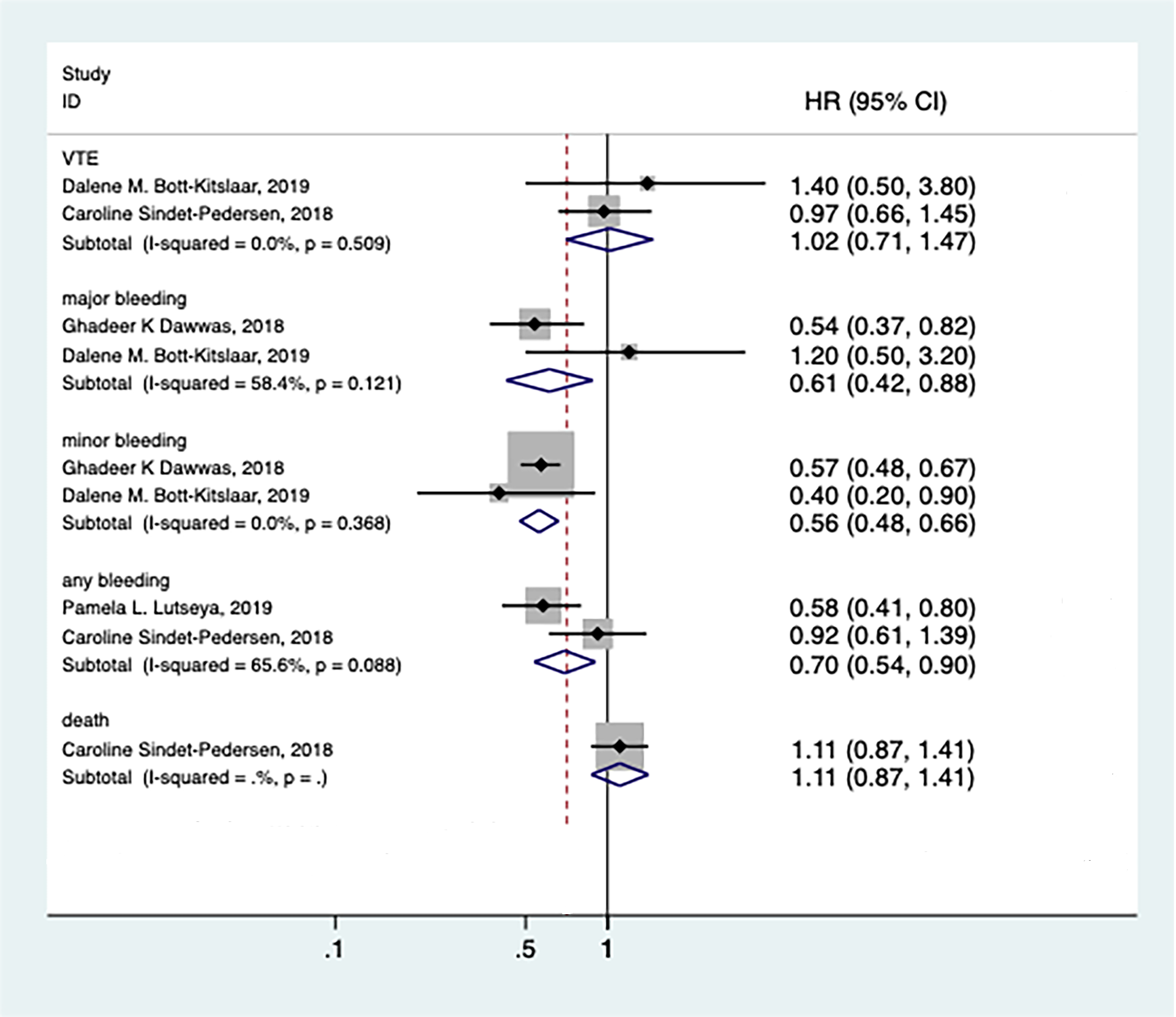

Supplement: Supplementary file 1 — Additional file 1. Figure S1. Comparison of apixaban and rivaroxaban in different clinical outcomes. [file 12872_2022_2550_MOESM1_ESM.tif]
