# Supplementary material for: Non-vitamin K antagonist oral anticoagulants in venous thromboembolism patients: a meta-analysis of real-world studies
Source: BMC Cardiovasc Disord. 2022 Mar 14;22:105. doi: 10.1186/s12872-022-02550-8 (PMC8922817; doi:10.1186/s12872-022-02550-8)
Supplement: Supplementary file 2 — Additional file 2. Definition of clinical outcomes in the study. [file 12872_2022_2550_MOESM2_ESM.docx]

Table S1 Definition of clinical outcomes in the study.

| Study | Outcomes | | Definition of clinical outcomes | |
| --- | --- | --- | --- | --- |
| Dawwas GK, 2018 ^[19]^ | Recurrent VTE, MB | | Recurrent venous thromboembolism was defined according to the presence of primary discharge diagnoses codes that have been validated previously and found to have a positive predictive value of 73-83%. Major bleeding events was defined according to ICD-9-CM. | |
| Bott-Kitslaar DM, 2019 ^[20]^ | Recurrent VTE, MB, CRNMB | | Major bleeding was defined as fatal bleeding, bleeding in a critical area (intracranial, intraspinal, intraocular, retroperitoneal, or pericardial), or either overt bleeding plus a decrease in the hemoglobin level of 2 g/dL or more (1.2 mmol/L) after the incident or transfusion of 2 units or more of packed red blood cells. | |
| Davis DO, 2017 ^[21]^ | Recurrence VTE, bleeding | | Diagnosis of recurrent VTE required radiographic confirmation. Significant bleeding was defined as a decrease in hemoglobin of 2 mg/dL or more, intracranial hemorrhage, gastrointestinal hemorrhage, or bleeding requiring transfusion of packed red blood cells. | |
| López-Núñez JJ, 2019 ^[22]^ | Recurrence VTE, MB | | Bleeding events were classified as “major” if they were overt and required a transfusion of two units or more of blood, or were retroperitoneal, spinal or intracranial, or when they were fatal. Fatal bleeding was defined as any death occurring within 10 days of a major bleeding episode, in the absence of an alternative cause of death. | |
| Lutsey PL, 2019 ^[23]^ | Hospitalized bleeding | | Hospitalized bleeding events were identified based on hospitalization discharge codes indicating intracranial hemorrhage, gastrointestinal bleeding and other major bleeding. | |
| Sindet-Pedersen C, 2018 ^[18]^ | | All-cause mortality, recurrent VTE, and hospitalized bleeding. | | Recurrent VTE was defined as an in-hospital admission, using only primary in-patient’s hospital admissions. Hospitalized bleeding was defined as an in-patient hospital admission with a bleeding diagnosis. Secondary safety outcomes included intracranial and gastrointestinal bleeding. |

VTE: venous thromboembolism, SE: systemic embolism, Pulmonary embolism: MB: major bleeding, GI: gastrointestinal, CRNMB: clinically relevant nonmajor bleeding, ICD-9: International Classification of Diseases version 9.
