# Supplementary material for: Non-vitamin K antagonist oral anticoagulants in venous thromboembolism patients: a meta-analysis of real-world studies
Source: BMC Cardiovasc Disord. 2022 Mar 14;22:105. doi: 10.1186/s12872-022-02550-8 (PMC8922817; doi:10.1186/s12872-022-02550-8)
Supplement: Supplementary file 3 — Additional file 3. Table S2. Baseline comparison between different two groups. [file 12872_2022_2550_MOESM3_ESM.docx]

Table S2 Baseline comparison between different two groups.

| Details | drugs | | Mean ± SD | 95% CI | | drugs | Mean ± SD | | 95% CI | |  | | p |
| --- | --- | --- | --- | --- | --- | --- | --- | --- | --- | --- | --- | --- | --- |
| Age | | apixaban | 63.6±4.35 | 56.7±70.5 | rivaroxaban | | 60.5±4.60 | 53.1±67.8 | |  | | 0.358 | |
|  |  | Total | 62.0±4.47 | 58.3±65.8 |  | |  |  | |  | |  |  |
| Gender | | apixaban | 52.6±6.45 | 42.4±62.9 | rivaroxaban | | 52.0±2.09 | 48.6±55.3 | |  | | 0.865 | |
|  |  | Total | 52.3±4.45 | 48.6±56.0 |  | |  |  | |  | |  |  |
| Renal disease | | apixaban | 10.8±5.67 | 1.78±19.8 | rivaroxaban | | 7.28±5.66 | -1.73±16.3 | | | | 0.413 | |
|  |  | Total | 9.04±5.57 | 4.38±13.7 |  | |  |  | |  | |  |  |
| Antiplatelet | | apixaban | 13.0±7.41 | 1.18±24.8 | rivaroxaban | | 11.3±7.04 | 0.05±22.5 | |  | | 0.747 | |
|  |  | Total | 12.1±6.80 | 6.47±17.8 |  | |  |  | |  | |  |  |

SD: standard deviation; One-way ANOVA was used for comparison between groups, and p value <0.05 was considered significant.
